# Review of Intraoperative Adjuncts for Maximal Safe Resection of Gliomas and Its Impact on Outcomes

**DOI:** 10.3390/cancers14225705

**Published:** 2022-11-21

**Authors:** Hani Chanbour, Silky Chotai

**Affiliations:** Department of Neurological Surgery, Vanderbilt University Medical Center, Nashville, TN 37209, USA

**Keywords:** glioma, glioblastoma, extent of resection, intraoperative modality, intraoperative imaging, awake craniotomy, general anesthesia, mapping, fluorescence, survival

## Abstract

**Simple Summary:**

Understanding the impact of intraoperative modalities in glioma surgery on the extent of resection (EOR), survival, and complications is vital to maximizing safe resection while preserving neurological function. A systematic literature search was performed to assess the impact of intraoperative modalities of glioma surgery, including one or a combination of the following: intraoperative magnetic resonance imaging (iMRI), awake/general anesthesia craniotomy mapping (AC/GA), fluorescence-guided imaging, or combined modalities. The heterogeneity in reporting the amount of surgical resection prevented further analysis. The studies reviewed indicated that these modalities significantly improved EOR but most often underreported Progression-free survival/overall survival (PFS/OS). Combining intraoperative modalities during the same brain glioma operation seems to have the highest effect compared to each modality alone.

**Abstract:**

Maximal safe resection is the mainstay of treatment in the neurosurgical management of gliomas, and preserving functional integrity is linked to favorable outcomes. How these modalities differ in their effectiveness on the extent of resection (EOR), survival, and complications remains unknown. A systematic literature search was performed with the following inclusion criteria: published between 2005 and 2022, involving brain glioma surgery, and including one or a combination of intraoperative modalities: intraoperative magnetic resonance imaging (iMRI), awake/general anesthesia craniotomy mapping (AC/GA), fluorescence-guided imaging, or combined modalities. Of 525 articles, 464 were excluded and 61 articles were included, involving 5221 glioma patients, 7(11.4%) articles used iMRI, 21(36.8%) used cortical mapping, 15(24.5%) used 5-aminolevulinic acid (5-ALA) or fluorescein sodium, and 18(29.5%) used combined modalities. The heterogeneity in reporting the amount of surgical resection prevented further analysis. Progression-free survival/overall survival (PFS/OS) were reported in 18/61(29.5%) articles, while complications and permanent disability were reported in 38/61(62.2%) articles. The reviewed studies demonstrate that intraoperative adjuncts such as iMRI, AC/GA mapping, fluorescence-guided imaging, and a combination of these modalities improve EOR. However, PFS/OS were underreported. Combining multiple intraoperative modalities seems to have the highest effect compared to each adjunct alone.

## 1. Introduction

Gliomas are the most common primary intracranial tumors in adult patients. The age-standardized incidence rate of gliomas was 4.7 per 100,000 person-years [1]. Around 8000 new cases are diagnosed annually in the United States [2]. Gliomas originate from the glial cells of the central nervous system and are graded from I to IV according to the World Health Organization (WHO) classification. The most common histologic types in adult patients include glioblastoma (GBM) (grade IV), astrocytic tumors (grade I-III), oligodendroglioma (grade II-III), and ependymomas (grade I-III) [3]. Among gliomas, GBM holds the poorest prognosis, with 2-year survival rarely exceeding 10% [4]. Maximal safe resection is the standard of treatment in the neurosurgical management of gliomas [5]. Enhancing cytoreduction and minimizing neurologic deficits can be especially critical when gliomas involve or approach eloquent regions of the brain [6]. Achieving a high extent of resection (EOR) while preserving functional integrity has been shown to improve surgical outcomes and overall survival (OS) [5,7].

Adjuvant modalities and technological enhancements have led to improved identification and visual discrimination of the tumor–brain margin to assist neurosurgeons in the operating room [8]. Intraoperative imaging techniques such as intraoperative magnetic resonance imaging (iMRI) and fluorescence imaging using 5-aminolevulinic acid (5-ALA) and sodium fluorescein have shown an increased EOR in lesions primarily amenable to subtotal resection (STR) [8,9]. Furthermore, when operating around eloquent areas, awake or asleep surgery with intraoperative neuromonitoring aids has enhanced the detection of safe corridors for tumor access and provided accurate real-time feedback regarding critical structures such as motor and language areas [10,11].

How these modalities differ in their effectiveness in increasing the EOR, survival benefit, and complication profile remains relatively understudied. Understanding each adjuvant technological advancement and its impact on surgical outcomes is vital to guide the surgeon’s choice of modality for enhanced surgical precision. The objective of this narrative review was to outline the current literature regarding the advances of intraoperative modalities in glioma surgery utilized to achieve optimal resection.

## 2. Materials and Methods

**Figure 1:**
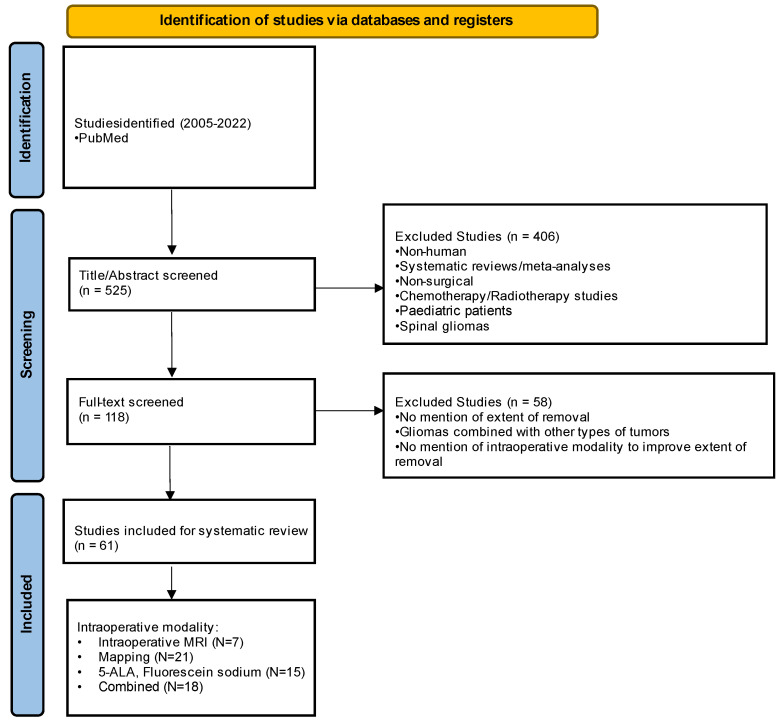
PRISMA flow of the systematic review.

An advanced PubMed search was performed in accordance with Preferred Reporting Items for Systematic Reviews and Meta-Analyses (PRISMA) guidelines (Figure 1) [12], using the following key search terms: “glioma”, “glioblastoma”, “GBM”, “MRI”, “monitoring”, “imaging”, “mapping”, “awake, “5-ALA”, “fluorescence”, “resection”, “adjunct”, and “removal”. No institutional review board approval was required since no original data were used. Inclusion criteria were studies published between 2005 and 2022, involving glioma surgery, and including one or a combination of intraoperative modalities such as iMRI, brain mapping through an awake craniotomy (AC) or general anesthesia (GA), or fluorescence-guided imaging. Exclusion criteria were non-human studies, review articles including narrative reviews, systematic reviews and meta-analyses, non-surgical management of gliomas, pediatric patients, spinal gliomas, case reports, and non-English language articles. Considering the rapid evolution of adjuncts used in glioma surgery over the last several years the studies prior to 2005 were excluded.

The primary outcome was the EOR, which was reported either as the percentage of resection, type of resection (i.e., gross total resection (GTR), subtotal resection (STR), etc.), or the amount of residual volume (cm^3^). Secondary outcomes were progression–free survival (PFS) and (OS), as well as postoperative complications. Postoperative transient neurologic deficits were not reported. Other collected variables included study period, study design, sample size, histologic diagnosis reported as GBM vs. lower-grade gliomas (I–II–III), and the location of tumors.

## 3. Results

Of 525 articles, 406 articles were excluded after title/abstract screening, and 568 were further excluded following the free-text screening. Of 61 remaining articles involving 5221 glioma patients, 7 (11.4%) articles used iMRI, 21 (36.8%) used cortical mapping, 15 (24.5%) used 5-aminolevulinic acid (5-ALA) or fluorescein sodium, and 18 (29.5%) used combined modalities (Figure 1).

### 3.1. Intraoperative Magnetic Resonance Imaging

In the early 1990s, neurosurgeons endorsed MRI technology in the operating room to improve tumor detection and enhance the resection of brain tumors [13]. iMRI assists neurosurgeons in demarcating the margins of the tumor and accounts for brain shifts on neuronavigation [14].

A total of 1170 patients were included in 4 prospective and 3 retrospective studies [15,16,17,18,19,20,21]. Moreover, 3/7 articles included lower-grade gliomas, and all 7 articles included patients with GBM. Studies that involved iMRI are summarized in Table 1.

In a retrospective study of 135 patients with GBM, Kuhnt et al. [17] identified residual tumors in 65% of cases using iMRI, which allowed further resection in 19 patients. Patients with GTR had an increased OS reaching 14 months compared to 9 months in the rest of the cohort, and 1 (0.7%) patient had a permanent language deficit. In a prospective study of 141 GBM and 83 (37.1%) lower-grade gliomas (I-III), Scherer et al. [16] performed an additional resection in 70% of patients after iMRI; however, 15 (6.7%) patients had a permanent new neurological deficit. While iMRI has demonstrated a significant EOR improvement in patients with GBM and lower-grade gliomas, the impact on PFS and OS was not well established. In a randomized controlled trial of 49 patients with GBM and lower-grade gliomas, Senft et al. [18] found a longer PFS in patients undergoing glioma resection under iMRI compared to the control group, but this did not reach statistical significance (*p* = 0.083). In another multicenter study, Shah et al. [21] found that while iMRI increased EOR, iMRI was not an independent predictor of OS.

Despite the increased operative time and the carried risk of infection and prolonged anesthesia, iMRI was demonstrated to be cost-effective with an incremental benefit of 0.18 quality-adjusted life years in high-grade gliomas [22].

### 3.2. Awake vs. Asleep Cortical and Subcortical Mapping

Intraoperative stimulation mapping was developed by Penfield and Cushing in the early 1900s [23] and is considered currently the gold standard in identifying eloquent areas of the brain. Mapping allows accurate real-time detection of areas with important functionality, in addition to tumor-related epileptic foci [24,25]. Mapping of eloquent areas is performed under either GA or AC protocol [26]. In AC, patients are asked to perform motor tasks to evaluate muscle strength and coordinate subcortical stimulation, which would ensure subcortical tract integrity prior to tumor resection.

As summarized in Table 2A–C, a total of 1961 patients were included in 3 prospective, 17 retrospective studies, and 1 combined prospective and retrospective study [26,27,28,29,30,31,32,33,34,35,36,37,38,39,40,41,42,43,44,45,46]. Of 21 articles using brain mapping, 18 articles included lower-grade gliomas, and 16 articles included patients with GBM.

GA mapping alone was used in 3 studies and included 346 patients (Table 2A). With regards to GA monitoring, Duffau et al. [38] found a higher total resection rate in patients undergoing lower-grade gliomas resection in patients with GA monitoring (25.4%) compared to patients without (6%), with no significant impact on OS.

AC mapping alone was used in 8 studies and included 404 patients (Table 2B). Motomura et al. [27] performed retrospective studies of 126 lower-grade gliomas using AC, and found an EOR of 93.1%, with 32 (25.4%) patients undergoing GTR. In another retrospective study 22 patients undergoing surgery for lower-grade gliomas, 11 (50%) of which underwent AC, EOR was higher in patients with monitoring vs. without (91.7% vs. 48.7%, *p* = 0.001), and OS was higher in patients receiving AC (PFS mean 5.3 vs. 3.7years, *p* = 0.008) [39].

Of 21 included studies, 9 studies included both GA and AC cohorts, involving a total of 1211 patients (Table 2C). While previous literature was in relative agreement regarding the established effect of AC and GA mapping on increased EOR, improved OS, and decreased postoperative morbidity, the superiority of one protocol against the other was hardly found in the literature, with not enough comparison of PFS, OS, and complications between patients with AC and GA monitoring [10,26,47,48].

### 3.3. Fluorescence-Guided Imaging

Under the operating microscope, the similarity between tumor appearance and nearby brain parenchyma makes it challenging to achieve a complete tumor resection. Fluorescence-guided surgery has provided neurosurgeons with real-time distinction of not only the tumor core, but also the tumor–brain interface that defines the extent of resection [49]. Biomarkers that were mostly investigated to maximize the extent of glioma resection are 5-ALA and fluorescein sodium.

A total of 937 patients were included in 10 prospective and 5 retrospective studies [50,51,52,53,54,55,56,57,58,59,60,61,62,63,64]. Of 15 articles using fluorescence-guided imaging, 6 articles included lower-grade gliomas, and 13 articles included patients with GBM. Studies that used fluorescence-guided imaging are summarized in Table 3A–C.

In 1947, fluorescein sodium was introduced to identify malignant gliomas. Fluorescein sodium is given intravenously, and it enters the brain in the areas of blood–brain barrier breakdown, where it resides in the extracellular space. Fluorescein sodium can be grossly perceived by the naked eye and allows real-time visualization of the areas contrasted by gadolinium in MRI [65]. Of 15 included studies, fluorescein sodium alone was used in 7 studies and included 275 patients (Table 3A). In a retrospective study of 48 patients with GBM, Catapano et al. [62] found a higher GTR (82.6%) in patients with sodium fluorescein-guided removal compared to controls (36%). Acerbi et al. [56] was the first group to initiate a phase II clinical trial in 20 patients undergoing high-grade glioma resection using sodium fluorescence and found that 80% of patients achieved a complete resection, with a sensitivity of 80% in detecting tumor tissue. In another prospective study conducted by Chen et al. [60], patients with fluorescein sodium showed a longer PFS compared to the control group (7.2 vs. 5.4 months). While previous studies investigated EOR and PFS in patients undergoing glioma removal with fluorescein sodium, the impact on OS remains understudied.

On the other hand, 5-ALA is ingested orally prior to surgery and is rapidly absorbed into the bloodstream and converted into protoporphyrin IX intracellularly. The latter molecule emits fluorescence when excited by blue-violet light conditions, facilitating the identification of tumor tissues [66]. Of 15 included studies, 5-ALA imaging was used in 7 studies and included 563 patients (Table 3B). In a phase III randomized controlled trial performed by Stummer et al. [53] involving 237 GBM and 85 lower-grade gliomas, patients who received 5-ALA had a higher GTR and PFS compared to the control group. Only one study utilized both 5-ALA and fluorescein sodium (Table 3C). When comparing 5-ALA and fluorescein sodium, Zeppa et al. [50] included 99 patients with GBM and found no significant difference in EOR or OS between 5-ALA alone, sodium fluorescein alone, and a combined 5-ALA sodium fluorescein-guided imaging.

### 3.4. Combined and Other Adjuvant Modalities

Combining intraoperative modality is not uncommon in glioma surgery. A total of 1153 patients were included in 4 prospective and 14 retrospective studies [67,68,69,70,71,72,73,74,75,76,77,78,79,80,81,82,83,84]. Of 18 included articles, 16 included lower-grade gliomas, and all articles included patients with GBM. A total of 10 studies used iMRI and mapping either through AC or GA, 5 studies used iMRI and 5-ALA fluorescence, 2 studies used 5-ALA fluorescence and mapping, and 1 study used iMRI and intraoperative tractography mapping. Studies that used combined intraoperative modalities are summarized in Table 4.

A few studies demonstrated that combining iMRI with AC or GA mapping may be more advantageous than either adjunct alone [69,70,71,72,73,74,75,76,77]. In 41 patients undergoing glioma resection with AC, Maldaun et al. [69] showed that 17 (40.5%) of cases underwent further resection after iMRI, with an increased EOR from 56% to 67%. Similarly, iMRI combined with 5-ALA has shown a potential increase in EOR and OS while keeping a safe complication profile [67,79,80,82]. Roder et al. [78], found a higher rate of GTR in patients undergoing iMRI versus those who received 5-ALA only in the surgical resection of GBM.

A combination of 5-ALA and GA with mapping was also reported [81,83]. In a prospective study of 36 patients with various grade gliomas undergoing surgical resection, Feigl et al. [81] found a 64% GTR rate when combining 5-ALA and GA with mapping. In another prospective study of 31 patients with low-grade and high-grade gliomas, GTR was achieved in 74% of patients when combining 5-ALA with either AC or GA with mapping [83]. These studies concluded that the combination of different intraoperative modalities seems to have a synergistic overall effect on EOR and overall survival.

Other modalities in adjunct to the aforementioned imaging include intraoperative or contrast-enhanced ultrasound. Ultrasound is cost-effective, widely available, offers real-time visualization of gliomas, and improves the accuracy of navigation. Additionally, doppler ultrasound tracks the flow patterns through the tumor’s microvessels using radiation-free images [85]. Prada et al. [86] found an increased EOR in patients undergoing GBM resection using contrast-enhanced ultrasound. Furthermore, in a retrospective study of 75 patients with GBM, Neidert et al. [87] found an increased OS and PFS with intraoperative ultrasound compared to the non-ultrasound group.

A few studies have described the use of intraoperative histology using stimulated Raman scattering microscopy, which was introduced in 2008 [8,88]. Artificial intelligence has shown high accuracy in differentiating high- and low-density tumor regions, detecting tumor subtypes, and distinguishing infiltrative cells at tumor margins through hand-held probes, which showed a potential improvement in EOR and subsequently PFS and OS. [89,90] Nonetheless, the number of studies reporting the use of intraoperative histology is limited.

## 4. Discussion

Considering the plethora of advancements in neurosurgery, we conducted a systematic review to summarize the recent investigations of the intraoperative modalities that assist the surgical treatment of gliomas, including iMRI, awake and asleep cortical and subcortical mapping, fluorescence-guided imaging, and combined modalities. While a number of studies have been published focusing on each adjuvant modality, studies comparing one adjuvant intraoperative modality with another are lacking.

The use of iMRI as an adjunct was associated with an increase in EOR; however, its impact on PFS and OS was limited. Though iMRI offers a vast range of advantages and intraoperative capabilities in improving EOR, it is limited by its feasibility and cost. Furthermore, special infrastructure requirements, as well as special expertise, are required to operate the use of iMRI [91].

Patients undergoing glioma removal under AC or GA monitoring have shown an increased EOR, PFS, and OS. However, the superiority of AC over GA remains to be elucidated. When considering AC, patient selection, appropriate patient selection, and coordination between experienced anesthesia, surgery, neuromonitoring, neurology, and neuropsychology teams are required. The risk of intraoperative seizures, postoperative emotional distress, and the need for conversion from AC to GA should be considered [26,32,39,92]. Regardless of the AC or GA approach, cortical and subcortical mapping of motor areas facilitates maximal safe resection. Studies have demonstrated that motor evoked potential (MEP) in motor mapping exhibits high reliability with few to no false-negative monitoring [93]. For the speech region and insular gliomas, AC with intraoperative language mapping is associated with improved EOR [26,94]. Studies comparing AC and GA for these eloquent regions are sparse.

While more studies have already established the significant impact of 5-ALA on EOR, PFS, and OS, research involving fluorescein sodium remains minimal. With regard to possible disadvantages of fluorescence-guided images, previous evidence showed an increased fluorescence in both low- and high-grade gliomas, some studies witnessed a lack of visible fluorescence in grade I and grade II gliomas, rendering high-grade gliomas more sensitive to fluorescent biomarkers [95]. Side effects of fluorescent agents include a sensitization of the skin that lasts for 24 h after ingestion. Furthermore, reactive astrocytosis, highly vascularized areas, and inflamed tissues may emit fluorescence regardless of the presence of malignancies [67]. Moreover, due to the lack of uptake of fluorescent biomarkers in non-vascularized tissues, the necrotic portion of the tumor may not be accurately detected [61]. Other downsides include the requirement of a dark surgical field, the cost associated with fluorescent biomarkers, and the time required for 5-ALA to reach peak concentration in blood [56,96].

Combining different intraoperative modalities is associated with a significant increase in EOR, PFS, and OS while keeping a safe complication profile. Cost-effectiveness, however, may play an important role in decision making. While other combined modalities have shown a greater effect compared to each surgical adjunct alone, randomized clinical trials are still lacking.

Newer advanced visualization technologies such as exoscope, contrast-enhanced ultrasound, intraoperative histopathology, imaging probe devices, stimulated Raman scattering microscopy, probe-based microscopy, and wide-field endomicroscopy are currently being tested and implemented intraoperatively [8]. Therefore, surgeons may employ the adjuncts based on their own expertise with various technologies, tumor histology and location, and cost-effectiveness. Other intraoperative modalities include fluorescence using indocyanine green, which demonstrated an increased affinity to endothelial cells in the peritumoral tissue [97], theranostics using optical coherence tomography that improved resection at a cellular-level precision [98], and other imaging modalities such as stereotactic navigation [99], whole-brain tractography, and diffusion tensor imaging [100].

This systematic review has some limitations that warrant further discussion. First, the allocated time for the search strategy (2005–2022), though arbitrary, aimed at removing non-relevant studies and narrowing the review of recent articles regarding intraoperative modalities. Second, the modalities that were explored in the listed studies may not be mutually exclusive. For example, some studies may have focused on iMRI but also have also used GA monitoring without mentioning it. Third, the lack of consistency in reporting EOR prevented further analysis. Fourth, while the current objectives were to report the PFS and OS in studies investigating intraoperative modalities for glioma resection, these parameters were underreported, likely due to the previously established correlation between EOR and prolonged survival, and some studies may have waived the need to explore this area of research. Finally, the heterogeneity of the location of gliomas was not accounted for in most of these studies, which might have impacted the EOR, survival analysis, and operative complications. The impact of postoperative adjuvant chemotherapy and radiotherapy on PFS and OS is not well reported in the studies reviewed. Furthermore, most studies investigating intraoperative modalities comprised small sample sizes. Large-scale multicenter studies are necessary to accurately establish the differences in EOR, PFS, and OS with the use of these adjuvant intraoperative modalities.

## 5. Conclusions

Intraoperative adjuncts such as iMRI, AC and GA mapping, fluorescence-guided imaging (5-ALA and fluorescein sodium), and a combination of these modalities have the potential to improve the extent of maximal safe resection for gliomas. The impact of using these adjuncts on PFS and OS is underreported. Combining multiple intraoperative modalities seems to have the highest effect compared to each adjunct alone. Future prospective studies with larger sample sizes are warranted to evaluate the differences between these modalities and their impact on short- and long-term outcomes.

## Figures and Tables

**Table 1:** Studies investigating the role of intraoperative MRI in improving the extent of resection.

Study (Years)	Study Period	Study Design	Tumors	Histology	Location	Extent of Resection	Overall Survival	Complications
Coburger et al. (2017) [15]	2008–2013	Retrospective	62	GBM	Non-specific	EOR 78 ± 4.0%	-	9 (27%) complications 2 (6%) CSF leak, 2 (6%) bleeding, 3 (9%) ischemia, 1 (3%) infection 1 (3%) hydrocephalus
Scherer et al. (2016) [16]	2011–2014	Prospective	224	141 (62.9%) GBM, 83 (37.1%) lower-grade gliomas	Frontal, temporal, parietal, occipital, intraventricular	70% underwent additional resection after iMRI	-	15 (6.7%) permanent neurological deficits
Kuhnt et al. (2011) [17]	2002–2008	Retrospective	135	GBM	Supratentorial	Residual tumor in 88 patients. Further resection was performed in 19 patients. GTR for 9 patients increased from 47 (34.8%) to 56 (41.5%) patients.	Mean OS in patients with GTR (EOR > 98%) = 14 months compared to 9 months in EOR < 98%)	1 (0.7%) permanent language deficit
Senft et al. (2011) [18]	2007–2010	Prospective	49	46 (93.9%) GBM, 3 (6.1%) lower-grade gliomas	Non-specific	GTR 23/24 (96%) in the iMRI group, and 17/25 (68%) in the control group.	Median PFS: 226 days (95% CI 0–454) in the iMRI group and 154 days (60–248) in the conventional group	5 (10.2%) permanent neurological deficits
Hatiboglu et al. (2009) [19]	2006–2007	Prospective	46	29 (63.0%) GBM, 17 (37.0%) lower-grade gliomas	Non-specific	EOR increased from 76% (range, 35%-97%) to 96% (range, 48%-100%) 29 patients had GTR, 15 (52%) due to iMRI	-	4/46 (9%) permanent neurological deficits (3 hemiparesis and 1 visual deficit)
Kubben et al. (2014) [20]	2010–2012	Prospective	14	GBM	Supratentorial	Residual tumor volume: control group median IQR (6.5%, 2.5–14.75%), iMRI group (13%, 3.75–27.75%)	iMRI: median (IQR) = 396 (191–599) days, control: 472 (244–619) days	-
Shah et al. (2020) [21]	1996–2008	Retrospective	640	GBM	Non-specific	Additional resection was performed in 104/122 with STR after iMRI,	Median OS was 17.0 months for patients with and without iMRI. iMRI was not associated with increased OS	Use of iMRI was not associated with increased rate of new permanent neurological deficit

**Table 2:** (**A**–**C**) Studies investigating the role of intraoperative mapping through awake and general anesthesia monitoring in improving the extent of resection: (**A**) general anesthesia; (**B**) awake craniotomy; (**C**) general anesthesia and awake craniotomy.

Study (Years)	Study Period	Study Design	Tumors	Histology	Location	Mapping	Extent of Resection	Overall Survival	Complications
(**A**): General Anesthesia									
Gogos et al. (2020) [19]	2018–2019	Prospective	58	37 (62.7%) GBM, 21 (37.3%) lower-grade gliomas	Frontal, temporal, parietal, insula	GA	Median EOR 98.0%	-	2 (3.4%) permanent neurological deficits
Schucht et al. (2014) [32]	2010–2014	Retrospective	67	GBM	Proximity to corticospinal tract	GA	Complete resection in 49/67 (73%)	-	3 (4%) persisting postoperative motor deficits
Duffau et al. (2005) [38]	1985–1996 and 1996–2003	Retrospective	222	Lower-grade gliomas	Eloquent areas	122 (54.9%) GA	Total resection: GA monitoring 31 (25.4%), no GA monitoring 6 (6%)	Survival: in GA monitoring: 11 (9.0%), non-GA monitoring: 43 (43%)	-
(**B**): Awake Craniotomy									
Motomura et al. (2021) [27]	2012–2020	Retrospective	126	Lower-grade gliomas	Frontal, insular, temporal, parietal, occipital	AC	Median EOR 93.1% >100% (=supratotal resection) 15 (11.9%) 100% (=gross total resection) 32 (25.4%) ≥90%, <100% (=subtotal resection) 27 (21.4%) <90% (=partial resection) 52 (41.3%)	-	5 (4.0%) permanent speech disturbance 9 (7.1%) permanent motor disturbance
Burks et al. (2017) [30]	2012–2015	Retrospective	15	11 (73%) GBM, 4 (26%) lower-grade gliomas	Non-specific	AC	100% (GTR) 12 (80%) 90%–99% (NTR)1 (7%) 70%–89% (STR) 2 (13%)	4 died (26.6%)	1 (7%) abulia 1 (7%) hemorrhage 1 (7%) Infection
Clavreul et al. (2021) [33]	2004–2019	Retrospective	46	GBM	Non-specific	AC	Complete resection in 28 (61%)	Median PFS was 6.8 months (CI 6.1; 9.7) and median OS was 17.6 months (CI 14.8; 34.1).	3 (6.5%) new motor deficits
Alimohamadi et al. (2016) [37]	2015–2016	Retrospective	10	3 (30%) GBM, 7 (70%) lower-grade gliomas	Non-specific	AC	73–100% EOR	-	1 (10%) deteriorated aphasia
Martino et al. (2013) [39]	2009–2011	Retrospective	22	Lower-grade gliomas	SMA, premotor, posterior temporal	11 (50%) AC	With monitoring: EOR (%) 91.7%, GTR 5 (45.5%), NTR 4 (36.4%), STR 2 (18.2%) without monitoring: EOR (%) 48.7%, GTR 0, NTR 4 (36.4%), STR 7 (63.6%),	5.3 years (3.5–7) monitoring 3.7 years (3.5–4) no monitoring	1 (9.1%) in AC (mild dysphasia), and 5 (45.5%) in no monitoring group (4 dysphasia, 3 hemiparesis).
Schucht et al. (2013) [42]	2007–2010	Retrospective	64	Lower-grade gliomas	Central, frontal	AC	Median EOR 92% (range 80–97%)	-	3 (9.1%) in central and 2 (6.5%) in frontal
Saito et al. (2019) [45]	2000–2018	Retrospective	30	3 (10.0%) GBM, 27 (90%) low-grade gliomas	Precentral gyrus	AC	EOR, Mean 93% (range = 75–100)	-	8 (26.6%) motor decline
Motomura et al. (2017) [44]	2012–2014	Retrospective	33	4 (12.1%) GBM, 29 (87.9%) lower-grade gliomas	Frontal, insular, parietal, temporal, occipital	AC	EOR ≥ 90% 15 (45.5%), <90% 18 (54.5%) increased rate of EOR due to iMRI in 16 patients by mean (SD) 15.8 (12.2)	-	3 (9.0%) permanent neurological deficits
Eseonu et al. (2018) [43]	2015–2016	Retrospective	57	17 (29.8) GBM, 40 (70.2%) low-grade gliomas	Peri-Rolandic motor area	AC (33 positive mapping (PM), 24 negative mapping (NM))	EOR 87.8% (7.1%) in positive monitoring, 92.4 (9.4%) in NM HG, positive monitoring: 85.7 (9.4%), HG, negative monitoring: 90.7 (10.2%). LG, positive monitoring: 90.8 (10.4%), LG, negative monitoring: 97.0 (5.2%)	-	3 (9.2%) in PM
(**C**): General Anesthesia and Awake Craniotomy									
Morsy et al. (2021) [28]	2014–2019	Retrospective	64	26 (40.6%) GBM, 48 (59.4%) lower-grade gliomas	Peri-Rolandic	40 (62.5%) AC, 24 (37.5%) GA	AC: EOR mean (SD) 92.03 (3.1) GTR > 98% 18 (45%) NTR > 90–98 14 (35%) STR 50–90% 8 (20%) GA: EOR, mean (SD) 90.05 (3.9) GTR > 98% 7 (29.2%) NTR > 90–98 8 (33.3%) STR 50–90% 9 (37.5%)	-	AC: 2 (5%), GA: 2 (8.3%) permanent neuro deficit
Nossek et al. (2011) [31]	2007–2009	Retrospective	55	22 (40%) GBM, 33 (60%) lower-grade gliomas	Frontal, parietal, temporal	35 (63.7%) AC, 20 (36.3%) GA	GTR 39 (71%) STR 16 (29%)	-	7 (12.7%) patients had varying degrees of permanent motor deficits
Rossi et al. (2021) [34]	2018–2019	Retrospective	135	High-grade gliomas 73 (35%), low-grade gliomas 48 (54%)	Peri-Rolandic	66 (49%) AC, 69 (51%) GA	95% mean EOR (94% vs. 96% AC)	-	-
	2018–2019	Prospective	52	High-grade gliomas 16 (31%), low-grade gliomas 34 (65%)	Peri-Rolandic	35 (67%) AC, 17 (33%) GA	97% mean EOR in both AC and sleep	-	-
Giamouriadis et al. (2020) [35]	2017	Prospective	48	26 (56.5%) GBM, 32 (43.5%) lower-grade gliomas	Frontal, insular, temporal, parietal, occipital.	16 (33.3%) AC, 32 (66.6%) GA	GA: GTR 27 (84.3%) STR 1 (3.1%) near GTR 3 (9.3%) AC: STR 0 near GTR 4 (25%) GTR 12 (75%)	-	GA: 1 (3.1%) permanent deficit AC: 2 (12.5%) permanent deficits
Eseonu et al. (2017) [47]	2005–2015	Retrospective	58	11 (18.9%) GBM, 47 (81.1%) lower-grade gliomas	Peri-Rolandic	27 (46.5%) AC, 31 (53.4%) GA	AC: mean (SD) EOR 86.3% (20.5%), GA: mean (SD) EOR 79.6% (23.1%)	-	-
Li et al. (2021) [48]	2008–2019	Retrospective	109	GBM	Primary motor cortex, primary sensory cortex, premotor cortex, language cortex	48 (44.0) AC, 61 (55.9%) GA	GA: mean (SD) EOR: 90.2% (7.44) Gross total (>95%), 28 (45.9) Subtotal (85–95%), 18 (29.5) AC: EOR: mean (SD) EOR: 94.9% (5.73) Gross total (>95%), 40 (83.3) Subtotal (85–95%), 6 (12.5) Partial (< 85%), 2 (4.2) Partial (< 85%), 15 (24.6)	AC: mean PFS 23.2 months mean OS 28.1 months GA: mean PFS was 18.9 months mean OS 23.4 months	Permanent motor 3 (9.7%) in AC vs. 3 (11.1%) in GA language deficit: 2 (6.5%) in AC vs. 4 (14.8%) in GA
Sacko et al. (2011) [40]	2002–2007	Prospective	575	120 GBM, 455 lower-grade gliomas	Frontal, temporal, parietal, occipital	214 (37.2%) AC, 316 (62.8%) GA	AC: 37% total, 45% subtotal. GA 52% total, 34% subtotal	-	Permanent neurological deficit: AC 20 (9.3%), and GA: 26 (8.2%)
Skrap et al. (2012) [41]	2000–2010	Retrospective	66	Lower-grade gliomas	Insular	46 (69.9%) AC, 23 (30.1%) GA	Median EOR 80% EOR 90%, n = 22 (33.3%) EOR 70%-90%, n = 30 (45.4%) EOR 70%, n = 14 (21.2%)	-	4 (6%) permanent deficits
Magill et al. (2018) [46]	1998–2016	Retrospective	49	15 (28.3) GBM, 34 low-grade gliomas	Primary motor cortex	34 (64.2%) AC, 19 (35.8%) GA	GTR 27 (50.9%) STR 26 (49.1%) Mean EOR 91% (range = 41–100)	-	20 (37.7%) permanent deficits

**Table 3:** (**A**–**C**) Studies investigating the role of intraoperative 5-ALA and sodium fluorescein in improving the extent of resection: (**A**) fluorescein sodium; (**B**) 5-ALA; (**C**) fluorescein sodium and 5-ALA.

Study (Years)	Study Period	Study Design	Tumors	Histology	Location	Modality	Extent of Resection	Overall Survival	Complications
(**A**): Fluorescein sodium									
Koc et al. (2008) [54]	2003–2006	Prospective	80	GBM	Non-specific	47 (58.7%) fluorescein sodium	GTR: 39 (83%) fluorescein sodium vs. 18 (55%) in control group.	No difference in median survival	-
Neira et al. (2017) [55]	2013–2014	Retrospective	32	High-grade gliomas (3–4)	Non-specific	Fluorescein sodium	27 (84%) GTR	-	-
Acerbi et al. (2018) [56]	2011	Prospective	46	High-grade gliomas	Non-specific	Fluorescein sodium	38 (82.6%) complete resections	PFS-6 and PFS-12 were 56.6% and 15.2%. Median survival was 12 months	-
Chen et al. (2012) [60]	2010–2011	Prospective	10	3 (30%) GBM, 7 (70%) lower-grade gliomas	Non-specific	Fluorescein sodium	8/10 (80%) GTR	7.2 PFS months vs. 5.4 in the control group	1/12 (8.3%) permanent hemiplegia in the control group
Diaz et al. (2015) [61]	-	Prospective	12	GBM	Non-specific	Fluorescein sodium	12/12 (100%) GTR	-	-
Catapano et al. (2017) [62]	2016–2017	Retrospective	48	GBM	Non-specific	23 (47.9%) fluorescein sodium	19/23 (82.6%) GTR vs. 9/25 (36%) in the control group	-	-
Francaviglia et al. (2017) [63]	2015–2016	Retrospective	47	33 (70.2%) GBM, 14 (29.8%) lower-grade gliomas	Non-specific	Fluorescein sodium	39/47 (83%)	-	6 (12.7%) hemorrhage with permanent hemiparesis 4 (8.5%) Seizures 1 (2.1%) Hydrocephalus 1 (2.1%) Sepsis
(**B**): 5-ALA									
Stummer et al. (2006) [53]	1999–2004	Prospective	322	237 (73.6%) GBM, 85 (26.4%) lower-grade gliomas	Non-specific	139 (43.1%) 5-ALA	GTR: 50 (36%) in 5-ALA group, 49 (27%) in the control group.	6-month PFS: 5-ALA: 57 (41.0%) vs. 39 (21.1%) in controls.	-
Nabavi et al. (2009) [51]	2003–2005	Prospective	36	21 (58.3%) GBM, 15 (41.7%) lower-grade gliomas	Non-specific	5-ALA	7/36 (19.4%) GTR	-	-
Diez Valle et al. (2011) [52]	2007–2009	Prospective	36	GBM	Non-specific	5-ALA	30 (83.3%) complete resections	PFS 6.5 months (95% CI 3.8–9.2) for newly diagnosed GBM, and 5.3 months (95% CI 4.4–6.2) for recurrent cases	-
Widhalm et al. (2010) [58]	2008–2009	Prospective	17	Lower-grade gliomas	Frontal, central, temporal, occipital, parietal, insular, thalamus	5-ALA	14/17 (82%) GTR	-	-
Widhalm et al. (2013) [57]	2008–2012	Prospective	59	Lower-grade gliomas	Frontal, central, temporal, occipital, parietal, insular, thalamus	5-ALA	38/59 (64%) GTR	-	-
Chan et al. (2018) [59]	2011–2016	Retrospective	16	10 (62.5%) GBM, 6 (37.5%) lower-grade gliomas	Non-specific	5-ALA	9/16 (56.2%) GTR	-	-
Teixidor et al. (2016) [64]	2010–2014	Prospective	77	66 (85.7%) GBM, 11 (14.3%) lower-grade gliomas	Non-specific	5-ALA	42 (54%) complete resections	Six-month PFS in 45 (58%) and median overall survival was 14.2 months	No serious adverse events were reported
(**C**): Fluorescein Sodium and 5-ALA									
Zeppa et al. (2022) [50]	2018–2021	Retrospective	99	GBM	Precentral, postcentral, temporo-insular	40 (40.4%) 5-ALA, 44 (44.4%) fluorescein sodium, 15 (15.2%) both	Total resection: 18/40 (45%%) 5-ALA, 21/44 (47.7%) in fluorescein sodium, and 6/15 (40%) in both	Mean (SD) OS 14.9 (9.91) months. mean OS in 5-ALA: 20 (16), SF: 12.3 (5.7), both 18.1 (11.9) months	-

**Table 4:** Studies investigating combined intraoperative modalities in improving the extent of resection.

Study (Years)	Study Period	Study Design	Tumors	Histology	Location	Modality	Extent of Resection	Overall Survival	Complications
Maldaun et al. (2014) [69]	2010–2011	Retrospective	41	9 (21.4%) GBM, 33 (78.6%) lower-grade gliomas	Frontal, temporal, parietal, insular	iMRI + AC	Median EOR overall was 90%, and gross total resection (EOR ≥ 95%) 17 (40.5%). After viewing the first MR images after initial resection, further resection was performed in 17 cases (40.5%); the mean EOR in these cases increased from 56% to 67% after further resection	-	Neurologic deficits 11 (26.2%)
Maesawa et al. (2010) [68]	2007–2008	Retrospective	28	Lower-grade gliomas	Proximity to corticospinal tract	iMRI, intraoperative tractography mapping	24 (85.7%) STR	-	1 (3.5%) permanent motor deficit
Zhuang et al. (2016) [70]	2011–2013	Retrospective	30	6 (20%) GBM, 24 (80%) lower-grade gliomas	Dominant insular lobe	iMRI, + AC or GA mapping	iMRI increased resection from 90 to 93% in all cases, and 88% to 92% in low-grade gliomas. The use of iMRI also resulted in an increase in the percentage of gross and near-total resection from 53% to 77%	-	3 (11%) permanent language, 2 (7.1%) motor deficits
Ghinda et al. (2016) [71]	2011–2015	Retrospective	106	25 (23.6%) GBM, 81 (76.4%) lower-grade gliomas	Frontal, parietal, temporal, insular	iMRI, + AC	Mean EOR 92%, complete resection was achieved in 64 (60.4%). 30 (28.3%) patients underwent further resection after initial iMRI scanning, with 10.1% increase in mean EOR	-	-
Leuthardt et al. (2011) [72]	2008	Retrospective	12	3 (25%) GBM, 9 (75%) lower-grade gliomas	Eloquent areas	iMRI, + AC	5 (41.6%) GTR, 2 (16.6%) NTR, and 5 (41.6%) STR	-	1 (8.3%) with worse outcome
Lu et al. (2013) [73]	2011	Retrospective	30	11 (36.6%) high-grade gliomas, 19 (63.3%) low-grade gliomas	Eloquent areas	iMRI, + GA mapping	Median EOR significantly increased from 92.5% (range, 75.1–97.0%) to 100% (range, 92.6–100%) 11 (36.6%) additional tumor removal	-	1 (3.3%) permanent deficit
White (2018) [74]	2001–2016	Retrospective	36	17 (47.2%) GBM, 19 (52.7%) lower-grade gliomas	Left hemisphere	iMRI, + AC	20 (55.6%) GTR, 19 (53%) further resections under iMRI	-	3 (8.3%) permanent deficits
Whiting et al. (2019) [75]	2010–2017	Retrospective	62	18 (29.0%) GBM, 43 (69.3%) lower-grade gliomas	Frontal, temporal, parietal, insular	iMRI, + AC	41 (85.4%) had additional resection due to MRI. Median EOR 98.5%	-	2 (3.2%) residual speech difficulty, and 2 (3.2%) permanent weakness postoperatively
Peruzzi et al. (2011) [76]	2006–2008	Retrospective	44	28 (63.6%) GBM, 16 (36.3%) lower-grade gliomas	Frontal, temporal, parietal, occipital	iMRI, + AC/GA mapping	GTR in all patients	-	-
Tuominen et al. (2013) [77]	-	Retrospective	40	10 (25%) GBM, 30 (75%) lower-grade gliomas	Frontal, temporal, parietal	20 (50%) iMRI + AC, 20 (50%) iMRI	GTR: 10 (50%) iMRI + AC 11 (55%) iMRI	-	1 (5%) permanent neurological deficit iMRI + AC 4 (20%) permanent neurological deficit iMRI
Roder et al. (2014) [78]	2010–2012	Retrospective	117	GBM	Non-specific	66 (56.4%) 5-ALA, 27 (23.0%) iMRI, 19 (16.2%) iMRI and 5-ALA	iMRI EOR 53%, 5 ALA combined with iMRI increased EOR	-	11 (9.4%) permanent severe deficits
Coburger et al. (2015) [79]	2012–2014	Prospective	116	GBM	Non-specific	59 (50.8%) iMRI (Group 1), 57 (49.2%) 5-ALA and MRI (Group 2)	mean EOR 97.4% (87–100) iMRI, 99.7% (97–100) iMRI + 5-ALA GTR: 27 (82%) iMRI, 33 (100%) iMRI + 5-ALA	Median PFS (CI95%)Group 1: 6 months (2.4–9.6), Group 2: 6 months (4.6–7.4) OS (CI95%) Group 1: 17 months (7.6–26.4) Group 2: 18 (15.2–20.8)	7 (21%) iMRI, 11 (27%) iMRI+ 5-ALA
Schatlo et al. (2015) [80]	2003–2011	Retrospective	200	166 (83%) GBM, 44 (17%) lower-grade gliomas	Non-specific	58 (29%) 5-ALA only, 55 (27.5%) iMRI + 5-ALA, 87 (43.5%) neither.	5-ALA enhanced the achievement of gross total resection. GTR 25 (45%) with iMRI vs. 43 (30%) without iMRi	Median overall survival 13.8 months in the non-iMRI group and 17.9 months in the iMRI group, with no effect on PFS	-
Feigl et al. (2010) [81]	2007–2009	Prospective	36	15 (41.6%) GBM, 21 (58.3%) lower-grade gliomas	Frontal, temporal, parietal, insular, cerebellar	5-ALA, GA mapping	16/25 (64%) GTR	-	2 (1%) hemiparesis, and 1 (0.5%) homonymous hemianopia
Tsugu et al. (2011) [82]	2005–2009	Retrospective	33	20 (60.6%) GBM, 13 (39.4%) lower-grade gliomas	Non-specific	23 (69.6%) 5-ALA, 10 (30.4%) 5-ALA + MRI	GTR: 6/11 (54.5%) 5-ALA only 4/10 (40%) 5-ALA + iMRI	-	-
Della Puppa et al. (2013) [83]	2011–2012	Prospective	31	25 (80.6%) GBM, 6 (19.4%) lower-grade gliomas	Insular, frontal, temporal, language area	5-ALA with GA (N = 25, 80.6%) or AC (N = 6, 19.4%) mapping	GTR 23/31 (74%)	-	1 (3%) severe morbidity
Yamada et al. (2015) [67]	2004–2007	Prospective	99	67 (67.6%) GBM, 32 (32.4%) lower-grade gliomas	Non-specific	5-ALA + iMRI	GTR 51/99 (52%)	-	-
Pichierri et al. (2019) [84]	2014–2018	Retrospective	92	28 (30.5%) GBM, 64 (69.5%) lower-grade gliomas	Frontal, temporal, occipital, parietal, insular, cerebellar	26 (28.3%) iMRI (G1), 20 (21.7%) iMRI + AC (G2), 46 (50%) control (G3)	Group 1: Grade 2 GTR 46%, Grade 3 GTR 57%, Grade 4 GTR 63%. Group 2: Grade 2 GTR 55%, Grade 3 GTR 66%, Grade 4 GTR 41%, Group 3: Grade 2 GTR 41%, Grade 3 GTR 30%, Grade 4 GTR 36%	-	Memory/cognition 1(4%) G1, 1(4%) G2, 5(10.8%) G3. Parietal syndrome 1 (5%) G2, Hemiparesis 2 (4.3%) G3, Dysphasia 2 (4.3%) G3
